# Disruption of O-Linked N-Acetylglucosamine Signaling in Placenta Induces Insulin Sensitivity in Female Offspring

**DOI:** 10.3390/ijms22136918

**Published:** 2021-06-28

**Authors:** Mackenzie Moore, Nandini Avula, Seokwon Jo, Megan Beetch, Emilyn U. Alejandro

**Affiliations:** 1 Department of Integrative Biology and Physiology, University of Minnesota Medical School, University of Minnesota, Minneapolis, MN 55455, USA; macmoore@umn.edu (M.M.); avula010@umn.edu (N.A.); joxxx057@umn.edu (S.J.); beet0013@umn.edu (M.B.); 2 Department of Surgery, University of Minnesota Medical School, University of Minnesota, Minneapolis, MN 55455, USA

**Keywords:** beta-cells, fetal programming, glucose homeostasis, islet, metabolism, O-GlcNAcylation, O-linked N-acetylglucosamine (GlcNAc) transferase, OGT, pancreas, placenta

## Abstract

Placental dysfunction can lead to fetal growth restriction which is associated with perinatal morbidity and mortality. Fetal growth restriction increases the risk of obesity and diabetes later in life. Placental O-GlcNAc transferase (OGT) has been identified as a marker and a mediator of placental insufficiency in the setting of prenatal stress, however, its role in the fetal programming of metabolism and glucose homeostasis remains unknown. We aim to determine the long-term metabolic outcomes of offspring with a reduction in placental OGT. Mice with a partial reduction and a full knockout of placenta-specific OGT were generated utilizing the Cre-Lox system. Glucose homeostasis and metabolic parameters were assessed on a normal chow and a high-fat diet in both male and female adult offspring. A reduction in placental OGT did not demonstrate differences in the metabolic parameters or glucose homeostasis compared to the controls on a standard chow. The high-fat diet provided a metabolic challenge that revealed a decrease in body weight gain (*p* = 0.02) and an improved insulin tolerance (*p* = 0.03) for offspring with a partially reduced placental OGT but not when OGT was fully knocked out. Changes in body weight were not associated with changes in energy homeostasis. Offspring with a partial reduction in placental OGT demonstrated increased hepatic Akt phosphorylation in response to insulin treatment (*p* = 0.02). A partial reduction in placental OGT was protective from weight gain and insulin intolerance when faced with the metabolic challenge of a high-fat diet. This appears to be, in part, due to increased hepatic insulin signaling. The findings of this study contribute to the greater understanding of fetal metabolic programming and the effect of placental OGT on peripheral insulin sensitivity and provides a target for future investigation and clinical applications.

## 1. Introduction

Maternal stress, malnutrition, and environmental factors are associated with placental insufficiency which contributes to metabolic programming and long-term health implications for the offspring through the process of fetal programming [1,2,3,4]. Placental signaling can be modified in response to maternal cues which impact the sensitive developing fetal organ systems and can be adaptive, but can also have long-term consequences and predispose the offspring to disease later in life [2,3,5]. Fetal growth restriction, which is most commonly due to placental insufficiency, has been associated with perinatal morbidity and mortality as well as an increased risk of type two diabetes and obesity in adulthood [6,7,8,9]. Placental insufficiency is multifactorial but ultimately the adaptive capabilities of the placenta become overwhelmed resulting in inadequate nutrient transport and oxygen delivery to the fetus, to which the developing pancreas is particularly sensitive [10,11,12,13,14,15,16]. The disruption of beta cell development and function has been identified as a possible explanation for the increased susceptibility to diseases involving glucose homeostasis that are associated with placental insufficiency [8,17]. Notably, impaired placental nutrient transport has been shown to have lifelong detrimental effects on beta cell growth and function in humans and animal models [8,15,16,18,19,20,21,22].

Alterations in the nutrient-sensing pathways are associated with placental insufficiency related to changes in cellular signaling and impaired placenta development [23,24,25,26,27,28]. O-linked N-acetylglucosamine transferase (OGT) is known to contribute to fetal programming through nutrient signaling pathways that monitor the nutrient supply and demand and subsequently regulate the placental transport capacity through effects on glucose and amino acid utilization [3,10,23,29,30,31]. O-GlcNAcylation is the post-translational modification that is catalyzed by OGT and cleaved by O-GlcNAcase (OGA) [32]. OGT activity is primarily responsive to glucose, fatty acids, and amino acid availability and flux through the hexosamine biosynthetic pathway [33]. Alternations in O-GlcNAcylation have been linked to an impairment of implantation, cell differentiation, trophoblast proliferation, and placenta growth [34,35,36,37,38,39,40]. OGT and OGA are highly expressed in the placenta and several key placental proteins are OGT targets, including the histone variant H2A and the hypoxia-inducible factor-1α with aberrant O-GlcNAcylation which results in reduced placental vasculature development and fetal growth restriction in mice [1,24,41,42,43,44]. OGT is an X-linked gene with evidence for sexually dimorphic expression in the placenta where males have a lower OGT basal expression. As such, males are more sensitive to maternal stress and the associated abnormal placental development and growth restriction [45].

Given the sensing capability of OGT, it can respond to nutrient availability and physiological stresses, such as hypoxia, with changes in glucose metabolism, transcription, translation, cell growth, and signaling as well as mitochondrial and endoplasmic reticulum functions [33,46,47,48]. OGT is able to respond differentially to the type, severity, and duration of the stressor, and therefore, signaling through this pathway may mediate the effects of placental pathology, maternal stress, and hyperglycemia during pregnancy on the offspring [1,35]. It has been previously demonstrated that a reduction in placental OGT affects the programming of the hypothalamic–pituitary–adrenal stress axis in adulthood in a sexually dimorphic manner; however, its role in the fetal programming of the metabolism and glucose homeostasis remains unknown [25].

We aimed to determine the long-term metabolic phenotype for offspring with reduced placental OGT. In the following study, OGT was specifically reduced in the placenta by targeting cells of trophoblast lineage utilizing the Cre-Lox system under the control of the Cyp19-Cre promoter gene that is expressed in trophoblast cells starting at E6.5 [49]. The efficiency and fidelity of Cyp19-Cre to the trophoblast lineage of the placenta without expression in the fetus has been previously validated [11]. Metabolic parameters, as well as glucose handling and insulin sensitivity, were evaluated in mice on a standard, normal chow diet (NC), and when faced with the metabolic challenge of a high-fat diet (HFD). The investigation into placental OGT could provide a greater understanding of fetal metabolic programming and identify potential therapeutic targets to improve fetal outcomes and mitigate the long-term metabolic consequences for offspring of gestational diabetes and placental insufficiency.

## 2. Results

### 2.1. Reduced Placental OGT in Female Offspring on a Normal Chow Diet Led to an Increase in Fasting Blood Glucose

In order to investigate the effect of placental OGT on glucose homeostasis in the adult offspring, we first evaluated the baseline metabolic phenotype of male OGTKO^Pl^ as well as female OGTHet^Pl^ and OGTKO^Pl^ offspring compared to the sex-matched littermate controls. The metabolic parameters were assessed over the first 12 weeks of life on a normal chow diet (NC). There was no difference in the post-weaning body weight (BW) for male and female mice from 5 to 12 weeks (Supplementary Figure S1A,A’). The BW for a different cohort of mice was obtained from week of life 1 to 4, which also demonstrated no difference in BW (Supplementary Figure S1B,B’). On the evaluation of the body composition by EchoMRI, there was no difference in lean or fat mass for male (Supplementary Figure S1C,D) or female mice (Supplementary Figure S1C’,D’) after 12 weeks of the NC. For males and females on the normal chow, placental OGT reduction did not appear to have an effect on body weight or composition in the adult offspring.

**Figure 1:**
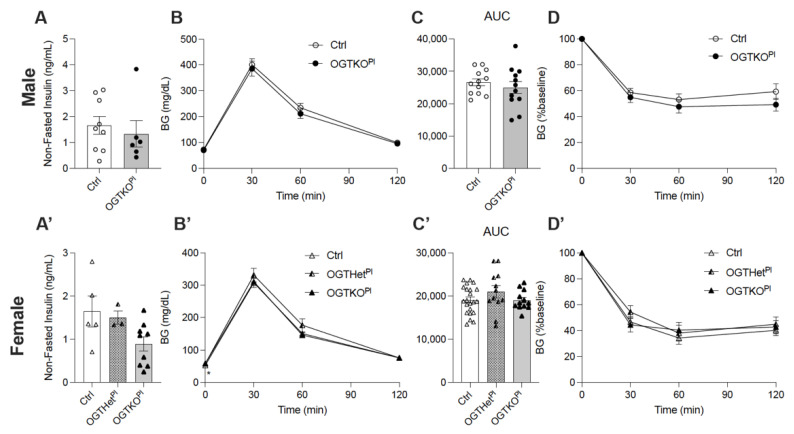
Reduced placental OGT in female offspring on normal chow had an increase in fasting BG. (**A**) Non-fasted insulin at 12 weeks of normal chow for male mice (n = 5, 7). (**A’**) Non-fasted insulin of female mice at 12 weeks of NC (n = 3, 9). (**B**) IPGTT and (**C**) calculated AUC for male (n = 12) and (**B’**,**C’**) female mice (n = 12, 22) at 6 weeks on normal chow. (**D**) ITT at 8 weeks on normal chow for male (n = 13, 14) and (**D’**) females (n = 12, 18). Blood glucose values post insulin injection are expressed as a percent of baseline blood glucose. (**A**,**C**) were presented as mean ± SEM, unpaired *t*-test for males and one-way ANOVA for females. (**B**,**D**) were presented as mean ± SEM, two-way ANOVA. *p* values, * *p* < 0.05.

Glucose handling was initially assessed by obtaining fasting blood glucose (BG) from mice on the NC at 6 weeks of age. The male control and the OGTKO^Pl^ mice had similar fasting BG levels (Supplementary Figure S1E). The non-fasted insulin levels at 12 weeks of age were not significantly different for males (Figure 1A). In contrast, the female OGTHet^Pl^ (*p* = 0.02) and OGTKO^Pl^ (*p* = 0.01) mice had a higher fasting BG compared to the controls (Supplementary Figure S1E’). The female OGTKO^Pl^ mice had lower non-fasted insulin levels at 12 weeks but this did not reach statistical significance (*p* = 0.06, Figure 1A’). Glucose homeostasis was further assessed with intraperitoneal glucose tolerance testing (IPGTT) and insulin tolerance testing (ITT) at 6 and 8 weeks of age, respectively. There were no notable differences in the IPGTT or the ITT between the male controls and the OGTKO^Pl^ mice (Figure 1B–D). For females, the OGTKO^Pl^ mice had a higher fasting blood glucose compared to the controls at T0 (Mean ± SD; 58.9 ± 4.8 vs. 52.7 ± 9.8, respectively) (*p* = 0.05, Figure 1B’,C’). There was no difference in the ITT across the female genotypes (Figure 1D’). On the NC, females with a reduction in placental OGT demonstrated an increase in fasting BG with a not statistically significant decrease in serum insulin.

### 2.2. High-Fat Diet-Induced Weight Gain Was Attenuated in Female Offspring with Reduced Placental OGT

**Figure 2:**
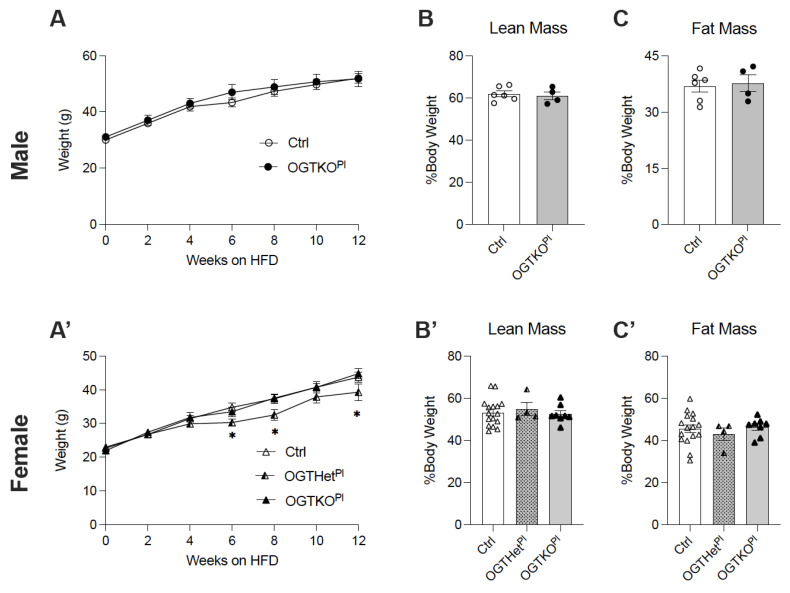
Placental OGT heterozygote females have decreased body weight starting at 6 weeks of HFD. (**A**) Body weight monitored across 12 weeks of HFD for males (n = 6, 7) and (**A’**) females (n = 10, 17). (**B**) Lean mass of males (n = 4, 6) and (**B’**) females (n = 4, 16) after 12 weeks HFD as assessed by EchoMRI. (**C**) Fat mass of males (n = 4, 6) and (**C’**) females (n = 4, 16) after 12 weeks HFD as assessed by EchoMRI. (**A**) was presented as mean ± SEM, two-way ANOVA. (**B**,**C**) were presented as mean ± SEM, unpaired *t*-test for males and one-way ANOVA for females. *p* values, * *p* < 0.05.

At 12 weeks of age, male and female mice were placed on a high-fat diet (HFD, 60% Kcal) to provide a metabolic challenge and to discern the differences in the response to this type of stressor. Both sexes gained weight and had an increase in adiposity as expected on an HFD treatment. The male control and the OGTKO^Pl^ mice similarly gained weight on the HFD (Figure 2A). Weight gain was similar for the female control and the OGTKO^Pl^ mice as well. Interestingly, the BW of the OGTHet^Pl^ female mice was reduced as compared to the female control and the OGTKO^Pl^ mice starting at 6 weeks and extending through the remainder of the time on the HFD (Figure 2A’). An altered placental OGT did not appear to affect lean or fat mass at 12 weeks of the HFD for either males (Figure 2B,C) or females (Figure 2B’,C’).

After 12 weeks of the HFD treatment, there was no difference in the pancreas, liver, and gonadal fat pad weight of male mice (Supplementary Figure S1F–H). Interestingly, female OGTHet^Pl^ mice had an increased pancreas weight in comparison to the controls and the OGTKO^Pl^ mice (*p* = 0.02 and *p* < 0.001, Supplementary Figure S1F’). A reduction in placental OGT did not appear to affect the liver and gonadal fat pad weight to BW ratio in females (Supplementary Figure S1F’–H’). 

### 2.3. No Changes in Food Intake or Energy Utilization in Female Mice with Reduced Placental OGT

**Figure 3:**
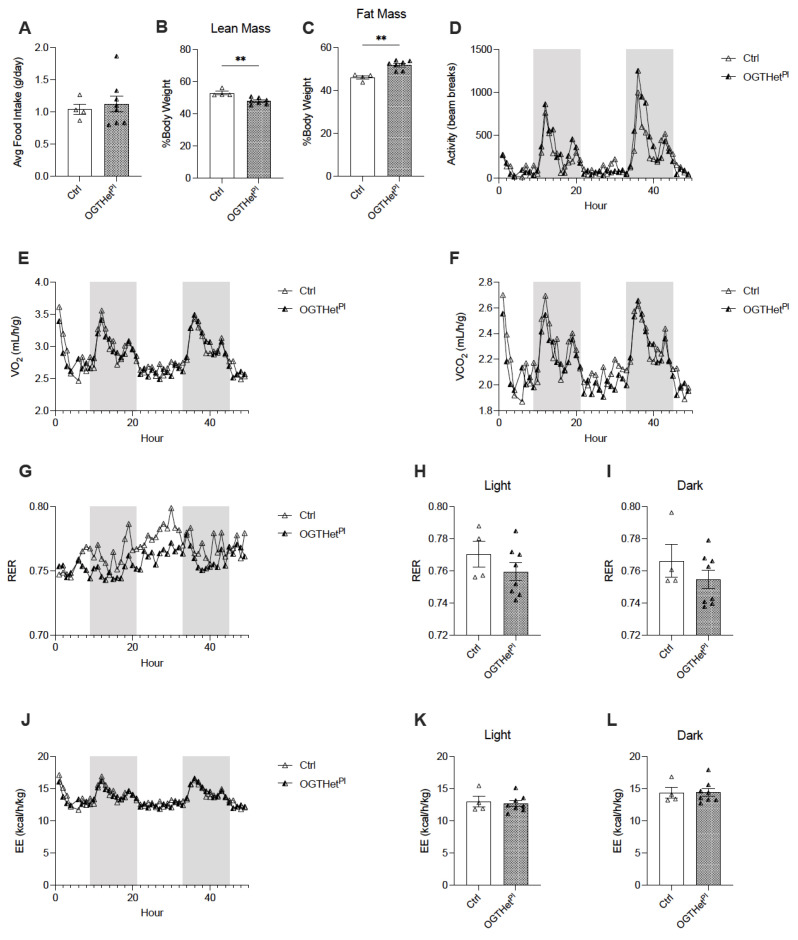
Increased fat mass and decreased lean mass for OGTHet^Pl^ female mice but no difference in energy balance on HFD. (**A**) Daily food intake of HFD averaged over three days for female mice (n = 4, 8). (**B**) Lean and (**C**) fat mass of females (n = 4, 7) after 16 weeks HFD as assessed by EchoMRI. (**D**) Activity evaluated by beam breaks, (**E**) Volume of oxygen (VO_2_) consumption, and (**F**) volume of carbon dioxide (VCO_2_) generation over 72 h (n = 4, 8) with 12 h light and dark cycles. Dark cycle designated by the shaded region. (**G**) Respiratory exchange ratio (RER) over 72 h (n = 4, 8) with 12 h light and dark cycles. Dark cycle designated by the shaded region. (**H**) Averaged RER for light and (**I**) dark cycles (n = 4, 8). (**J**) Energy expenditure (EE) over 72 h (n = 4, 8) with 12 h light and dark cycles. Dark cycle designated by the shaded region. (**K**) Averaged EE for light and (**L**) dark cycles (n = 4, 8). (**A**–**C**,**H**,**I**,**K**,**L**) was presented as mean ± SEM, unpaired *t*-test. (**D**–**F**,**G**,**J**) were presented as mean, two-way ANOVA. *p* values, ** *p* < 0.01.

In order to determine the etiology of the BW phenotype for the OGTHet^Pl^ female mice on the HFD, a separate cohort was continued on a HFD for an evaluation of food intake and energy utilization. The daily food intake demonstrated no difference in mice with reduced placental OGT compared to the controls at 16 weeks of the HFD (Figure 3A). The body composition of the OGTHet^Pl^ mice in this cohort was significant for decreased lean mass (*p* = 0.004, Figure 3B) and increased adiposity (*p* = 0.001, Figure 3C). For this cohort, the BW between the OGTHet^Pl^ mice and the control at 16 weeks of the HFD was not statistically different (Supplementary Figure S2A).

Next, the activity levels and energy utilization were assessed via indirect calorimetry and there was no apparent effect of a reduced placental OGT on the activity levels (Figure 3D). Furthermore, O_2_ consumption and CO_2_ production were comparable (Figure 3E,F), which resulted in similar measurements of fuel utilization as determined by the respiratory exchange ratio (RER) (Figure 3G). The RER was not significantly affected by the light/dark phase (Figure 3H,I). In addition, the energy expenditure did not differ between the OGTHet^Pl^ mice and the littermate controls (Figure 3J–L). 

### 2.4. Heterozygote Placental OGT Female Offspring Demonstrate Increased Insulin Tolerance on HFD

Fasting BG increased in a time-dependent manner over the course of 12 weeks on the HFD treatment for both sexes. Fasting BG for the male OGTKO^Pl^ mice was not significantly different from the littermate controls at all time points tested (Supplementary Figure S2B). In contrast to the data for mice on a normal chow, the fasting blood glucose of the OGTHet^Pl^ females was lower than the controls (*p* = 0.11) and OGTKO^Pl^ (*p* = 0.09) at 8 weeks of the HFD but did not reach the threshold for significance (Supplementary Figure S2C). Additionally, fasting insulin (Supplementary Figure S2D,E) and non-fasted blood glucose (Supplementary Figure S2F,I) did not differ between the genotypes for either sex over the course of the HFD treatment.

**Figure 4:**
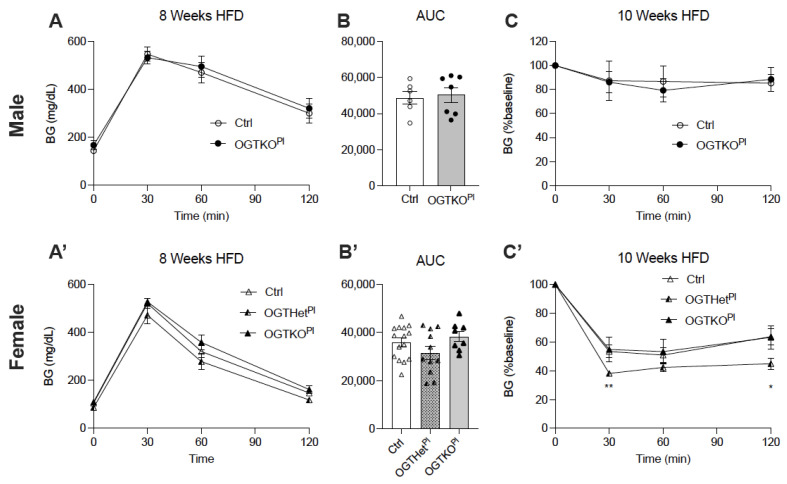
Heterozygote placental OGT female mice demonstrated increased insulin tolerance. (**A**) IPGTT and (**B**) calculated AUC at 8 weeks of HFD for male (n = 6, 7) and (**A’**,**B’**) female mice (n = 8, 15). (**C**) ITT at 10 weeks on HFD for male (n = 4, 5) and (**C’**) females (n = 7, 15). Blood glucose values post insulin injection are expressed as a percent of baseline blood glucose. (**A**,**C**) were presented as mean ± SEM, two-way ANOVA. (**B**) was presented as mean ± SEM, unpaired *t*-test for males and one-way ANOVA for females. *p* values, * *p* < 0.05, ** *p* < 0.01.

A difference in glucose homeostasis was suspected given the reduced BW and a non-significant lower fasting BG in the OGTHet^Pl^ mice, so this was further investigated. Over the course of the HFD treatment, both sexes developed a progressive glucose intolerance, as determined by the IPGTT at 4, 8, and 12 weeks. There was no difference in the degree of glucose intolerance between the genotypes for either sex (males, Figure 4A,B, Supplementary Figure S2J–M; females, Figure 4A’,B’, Supplementary Figure S2N–Q). Male mice did not demonstrate a difference in insulin tolerance between the controls and the OGTKO^Pl^ as assessed by the ITT at 6 weeks and 10 weeks of the HFD (Figure 4C, Supplementary Figure S2R). Female OGTHet^Pl^ mice exhibited an increased insulin tolerance in comparison to the control and the OGTKO^Pl^ mice at both time points (Figure 4C’, Supplementary Figure S2S). 

### 2.5. Female Placental OGT Knockout Offspring on HFD Demonstrated Impaired Insulin Secretion in Response to Glucose and No Change in Beta Cell Mass

**Figure 5:**
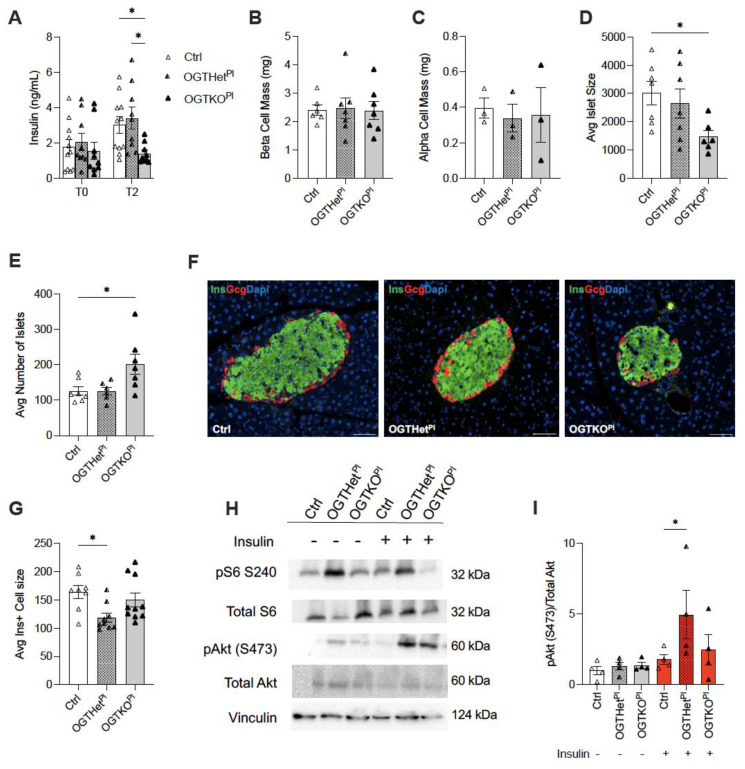
Increased pS473 (pAKT) in the liver of OGTHet^Pl^ mice in response to insulin treatment after 12 weeks HFD. (**A**) In vivo glucose stimulated insulin secretion (GSIS) for females (n = 9, 11). T0 is a fasted state. T2 is two minutes post insulin injection. (**B**) beta cell mass in females after 12 weeks on HFD (n = 7). (**C**) ⍺ cell mass in females after 12 weeks on HFD (n = 3). (**D**) Average islet size for females after 12 weeks on HFD (n = 7). (**E**) Average number of islets for females after 12 weeks on HFD (n = 7). (**F**) Representative islets from each female genotype post HFD for 12 weeks with insulin (green), glucagon (red), and DAPI (blue) immunostaining. Magnification 20×, scale bars 50 µm. (**G**) Average islet cell size for females after 12 weeks on HFD (n = 8, 10). (**H**) Western blot for pAKT (pS473), total Akt, pS6 (S240), and total S6 in the liver following injection of insulin or injection of PBS as a control. Samples were taken from females on 12 weeks HFD (n = 4). (**I**) Quantification of western blot for pAKT (pS473)/total Akt from liver samples visualized in (**G**). (**A**) was presented as mean ± SEM, two-way ANOVA. (**B**–**E**,**G**,**I**) were presented as mean ± SEM, one-way ANOVA. *p* values, * *p* < 0.05.

Because the glucose tolerance was normal despite the improvement in insulin tolerance, we speculated that the pancreatic endocrine function and mass were altered in the OGTHet^Pl^ females. To assess the contribution of the beta cell function in the insulin sensitivity we observed in the OGTHet^Pl^ mice, we performed an in vivo glucose stimulated insulin secretion (GSIS) at 12 weeks of the HFD. Both the control and the OGTKO^Pl^ males had a severely blunted insulin secretion in response to glucose after 12 weeks of the HFD treatment, which was expected (Supplementary Figure S3A). In comparison to the control, the OGTHet^Pl^ females displayed comparable insulin levels (Figure 5A). However, the OGTKO^Pl^ mice had an attenuated insulin secretion in response to glucose stimulation in comparison to the littermate control and the OGTHet^Pl^ females (Figure 5A). 

Previous work has found that fetal programming can contribute to the establishment of beta cell mass [17,18,21,22]. Therefore, endocrine cell masses were quantified and found to be similar across the female genotypes for beta cell mass (Figure 5B) and alpha cell mass at 12 weeks of the HFD (Figure 5C). The average islet size for the OGTKO^Pl^ mice was decreased compared to the controls (*p* = 0.04) but was compensated for by an increase in the total number of islets (*p* = 0.04, Figure 5D,F). Unexpectedly, the average insulin positive islet cell size was decreased in the OGTHet^Pl^ mice compared to the controls (*p* = 0.02) and the OGTKO^Pl^ mice (*p* = 0.11), but the later comparison did not reach statistical significance. (Figure 5G). 

### 2.6. Increased Insulin Sensitivity for OGTHet^Pl^ Females Was Associated with Increased Hepatic Insulin Signaling

The next aim was to assess the peripheral factors contributing to the increased insulin sensitivity in the OGTHet^Pl^ females on the HFD. Following 12 weeks of HFD, female mice were randomized to acute treatment with insulin or saline prior to tissue collection. Insulin signaling was assessed via a western blot of pAkt(S473) and total Akt. There was an increase in the hepatic pAkt/Total Akt ratio in the OGTHet^Pl^ mice treated with insulin, indicating an increased responsiveness and downstream signaling (*p* = 0.02, Figure 5H,I). The total Akt protein was comparable between the two groups (Supplementary Figure S3B. mTORC1 activation, measured by pS6/S6, was also not different between the genotypes (Supplementary Figure S3C). The total S6 demonstrated no differences (Supplementary Figure S3D).

## 3. Discussion

Adverse events in fetal development and the neonatal period have been associated with changes in susceptibility to metabolic disease for the offspring [8,18,19,20,21,22]. Nutrient-sensing pathways in the placenta have been implicated in mediating the effects of prenatal stress and fetal programming [23,24,25,26,27,28]. We aimed to establish the metabolic phenotype in the adult offspring with placenta-specific alterations in the nutrient-sensing protein, OGT.

In our study, glucose homeostasis appeared unaffected in males with a knockout of placental OGT on a normal chow diet and when faced with the metabolic challenge of a HFD. Female offspring with a reduced placental OGT demonstrated an improved insulin tolerance on a HFD, which correlated with an increased hepatic insulin/Akt signaling response. However, there was no difference in glucose tolerance in vivo or the total beta cell mass among the females on the HFD except for a diminished insulin secretion in response to glucose in the OGTKO^Pl^ mice. These data indicate a potentially protective phenotype for OGTHet^Pl^ females against HFD-induced weight gain and insulin resistance. 

Fetal programming is an adaptive mechanism that alters the gene expression in response to conditions during fetal development. Although adaptive at the time, this programming can predispose the offspring to obesity and metabolic disease later in life [50]. Fetal growth restriction and maternal malnutrition have been linked to the adult offspring’s increased risk of obesity and an impaired insulin sensitivity in humans and animal studies [51,52,53]. This could be related to the programming of glucose and insulin response in peripheral tissues such as adipocytes, hepatocytes, and skeletal muscle as well as central energy homeostasis mechanisms [52,54]. Our data show that the placental OGT was sufficient to impact insulin tolerance in the adult offspring. Thus, future studies can assess the source of insulin sensitivity in the female OGTHet^Pl^ offspring and determine whether insulin sensitivity is secondary to the lack of weight gain in response to a high-fat diet. 

A decreased placental OGT expression has been found in animal models to be a marker of prenatal stress and growth restriction [1,55]. A study of the targeted reduction in placental OGT via transgenic manipulation resulted in long-term altered neurodevelopment that mimicked models of prenatal stress. OGT appeared to affect gene expression in this study via histone modifications and thus contributed to the programming of the nervous system. The reduction in placental OGT resulted in an increased hypothalamic–pituitary–adrenal stress axis response in males and a decreased response in females [25]. Additionally, there was a dose–dependent decrease in BW with the reduction in placental OGT. In our study, a difference in BW on the NC was not observed. This could be due to different experimental exposures of stress and glucose homeostasis testing that affected the BW. Moreover, it could be explained by the mixed background of the animals or local differences in housing and environment such as microbiome differences.

Fetal programming of glucose homeostasis has been shown to occur through alterations in peripheral insulin sensitivity and effects on beta cell function. Adult offspring of dams on low-protein diets during pregnancy have been shown to have increased levels of insulin receptors on hepatocytes and skeletal muscle that were associated with enhanced insulin sensitivity through increases in GLUT-2 and GLUT-4, respectively [54,56]. Akt is downstream of the insulin receptor and promotes glucose uptake through GLUT4 and glycogen synthesis [57]. The present study observed increased hepatic insulin/Akt signaling in OGTHet^Pl^ females that could be contributing to an increased insulin sensitivity consistent with previous programming studies [58,59]. 

Additional studies into the effects of maternal malnutrition have identified defects in beta cell mass and function in response to a second challenge such as a HFD-induced glucose and insulin intolerance [60]. Beta cell mass compensation in response to the HFD challenge appeared unaffected by the reduction in placental OGT but the distribution of islets did differ, and glucose stimulated insulin secretion was decreased in the OGTKO^Pl^ female mice. Further investigation into beta cell function at the islet level may be revealing. 

The inactivation of the X chromosome in the placenta is more malleable than in somatic tissue and can be altered in a non-random fashion in response to maternal and fetal conditions [61]. This adaptability may partly account for the sex differences observed in response to prenatal stressors where females have shown to be protected compared to males [3]. Specifically, in our model, the OGTHet^Pl^ female mice may have had greater flexibility to increase or decrease OGT expression and subsequent O-GlcNAcylation flux which manifests as programmed protection from HFD-induced insulin resistance. 

The improved insulin tolerance of the OGTHet^Pl^ mice was consistent with prior investigations of maternal malnutrition and fetal programming but they did not develop glucose intolerance as seen in those previous studies [8,54,56]. Interestingly, there was no difference in the susceptibility to metabolic disease in OGTKO^Pl^ males that would resemble prenatal stress programming. The outcomes of programming can be specific to the insult or condition such that maternal diet changes and genetic manipulation of the placenta may yield distinct consequences for the offspring. Further, there can be differential, tissue-specific effects as well as sexually dimorphic outcomes in response in the programmed offspring. Identifying the mechanisms underlying the sexual dimorphism in this model is an important future direction.

The results of this study indicate that placental OGT manipulation can affect long-term responses to metabolic challenges through changes in peripheral insulin sensitivity. Continued work is warranted to gain a greater understanding of fetal metabolic programming and to investigate the potential clinical applications that would improve fetal outcomes for at risk pregnancies and address the programming that becomes maladaptive later in life.

## 4. Materials and Methods

### 4.1. Generation of Mice

The following mice were used in the current study: the OGT^flox/flox^ mice (purchased from Jackson Laboratories, B6.129-Ogttm1Gwh/J, Stock No: 004860), and the Cyp19-Cre mice were a gift from Dr. Gustavo Leone (The Ohio State University) [11,49,62]. Reduced placental OGT mice with the genotype of Cyp19-Cre; Ogt^flox/+^, and Cyp19-Cre; Ogt^flox/flox^ (herein referred to as OGTHet^Pl^ and OGTKO^Pl^, respectively, for females) and Cyp19-Cre, Ogt^flox/y^ (herein referred to as OGTKO^Pl^ for males) were generated by breeding Cyp19-Cre; Ogt^flox/+^ females with Ogt^flox/y^ males. Cyp19-Cre negative littermates were used as controls. Experimental animals were genotyped before weaning. All mice were generated on a mixed background and group housed on a 14:10 light-dark cycle with ad libitum access to a standard diet or a high-fat diet (HFD, D12492, 60% kcal fat, Research Diet Inc., New Brunswick, NJ, USA) starting at 12 weeks of age where indicated. For rigor, sex was considered as an independent variable and the data were segregated and analyzed separately. The sex of the mice is indicated in the figure legend and/or on the graph labels. All studies were approved by the Institutional Animal Care and Use Committee (protocol #1806-36072A) at the University of Minnesota.

### 4.2. In Vivo Mouse Studies

Body weight and non-fasted blood glucose were collected every two weeks unless otherwise indicated. Fasting blood glucose was obtained at 6 weeks of normal chow and every 4 weeks on the HFD. A blood sample for glucose measurement was obtained from the tail vein with a handheld glucometer. Facial vein blood was collected at 12 weeks for the normal chow and 12 weeks for the HFD as well as for glucose-stimulated insulin secretion experiments where indicated. Blood was collected into anticoagulant-coated or microcapillary tubes and centrifuged to obtain serum. This was stored frozen at −80 °C then assessed for insulin concentration using an ALPCO rat insulin ELISA, per the kit instructions. The analysis was completed by a 5-parameter logistic fit utilizing the MyAssays software.

Body composition (Echo-MRI, Echo Medical Systems LLC, Houston, TX, USA) and indirect calorimetry (Oxymax/CLAMS Lab Animal Monitoring System, Columbus Instruments) were conducted by the Integrative Biology and Physiology (IBP) Core at the University of Minnesota. Daily food intake was determined over 3 days by weighing the chow for single-caged mice.

Glucose tolerance testing (IPGTT) was completed by fasting the mice overnight (12 h) and measuring fasting blood glucose and then administering a 2 g/kg intraperitoneal injection of a 50% dextrose solution (D50). Post injection blood glucose was collected at 30, 60, and 120 min. Insulin tolerance testing (ITT) was assessed following a 6 h fast and blood glucose measurements were collected before and after (30, 60, and 120 min) a 0.75 U/kg dose of insulin in 0.9% saline (0.1 U/mL, Humalog, Lilly) was administered via intraperitoneal injection. Glucose-stimulated insulin secretion (GSIS) was evaluated by obtaining insulin levels from facial vein serum samples collected after an overnight fast and subsequently 2 min after a 3 g/kg intraperitoneal injection of D50 as previously described [63].

### 4.3. Histological Analysis

Pancreata, liver, and gonadal fat pads were freshly harvested, weighed, and fixed overnight in 3.7% formalin. They were then transferred to 70% ethanol at 4 °C until tissue processing. Pancreatic tissues were embedded in paraffin and we analyzed 5 series of 5 μm thick slices every 200 μm through the depth of the pancreas. Sections for immunofluorescence staining were deparaffinized and underwent antigen retrieval. These sections were then incubated with primary antibodies insulin (Sigma-Aldrich, Darmstadt, Germany, I6136) and glucagon (Abcam, ab10988) overnight at 4 °C. After washing, the sections were incubated with TexasRed- or FITC-conjugated secondary antibodies and DAPI. A Nikon Eclipse Ni-E microscope was used to obtain fluorescent images. Beta cell and alpha cell mass were calculated by determining the ratio of insulin-positive and glucagon-positive areas over the total pancreas area, respectively (quantified in FIJI), from 5 sections per pancreas and this ratio was multiplied by the pancreas weight as previously described [63]. Islet size, cell count, and size were quantified for these samples as well using FIJI. 

### 4.4. Acute Insulin Treatment In Vivo

Following a four hour fast, a subset of mice was randomized to intraperitoneal injection of 1 U/kg insulin or an equivalent volume of saline as a control. After 15 min, the mice were euthanized with CO_2_ [64,65]. Liver, skeletal muscle, and gonadal fat were preserved in liquid nitrogen for future western blot analysis. Western blot was performed by placing 50 μg of protein lysates in a RIPA buffer + 1% SDS (Bio-Rad) + protease and phosphatase inhibitors (CST), followed by Pierce BCA protein quantitation (ThermoScientific, Waltham, MA, USA), resolved by SDS-PAGE, and transferred to polyvinylidene difluoride membrane as previously described [66]. The primary antibodies utilized were: Phospho-Akt (S473) (Cell Signaling Technologies, 4060S), Akt-pan (40D4) (Cell Signaling Technologies, 2920S), Phospho-S6 (S240/244), (Cell Signaling Technologies, 5364S), S6 ribosomal protein (Cell Signaling Technologies, 2317S), and Vinculin (Cell Signaling Technologies, 13901S). The blot was visualized with SuperSignal West Pico PLUS (ThermoScientific), as per the manufacturer’s protocol. Densitometry analysis was performed with NIH ImageJ software. Values were normalized to the PBS control group.

### 4.5. Statistical Analysis

Data are presented as mean ± SEM. Male data were analyzed using an unpaired, two-tailed *t*-test. Female data from the three genotypes were analyzed using one-way ANOVA with Tukey post hoc test. Food intake and body composition data from the female cohort on 16 weeks of the HFD were analyzed using an unpaired, two-tailed *t*-test given that only two genotypes were studied. Repeated data measures for both sexes were analyzed using two-way ANOVA with the Dunnett post hoc test. Statistical analyses and visualization were performed in GraphPad Prism version 9 with a significance threshold of *p* < 0.05.

## Data Availability

Data available at request.

## Supplementary Materials

The following are available online at https://www.mdpi.com/article/10.3390/ijms22136918/s1. Figures S1–S3, Glucose homeostasis phenotypes in adult mice and western blot analysis of insulin treatment.
