# Supplementary material for: Disruption of O-Linked N-Acetylglucosamine Signaling in Placenta Induces Insulin Sensitivity in Female Offspring
Source: Int J Mol Sci. 2021 Jun 28;22(13):6918. doi: 10.3390/ijms22136918 (PMC8267851; doi:10.3390/ijms22136918)
Supplement: Supplementary file 1 [file ijms-22-06918-s001.zip › ijms-1254377-supplementary.pdf]

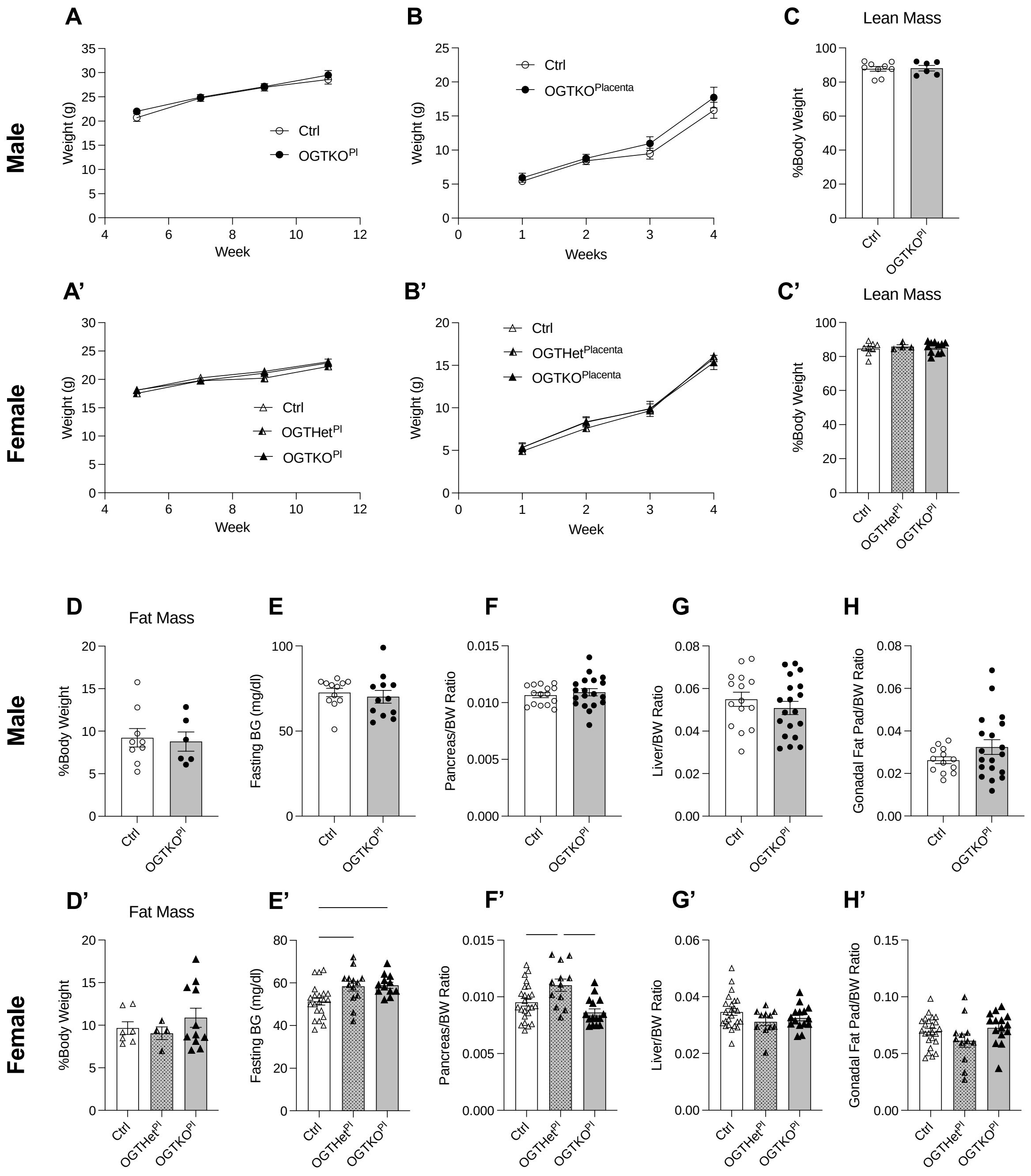

Supplementary Figure S1. (**A**) Body weight of males and (**A’)** females over 12 weeks on NC (n=12). (**B**) Body weight of males (n=6) and (**B’**) females (n=4, 8) over week of life 1-4. (**C**) Lean mass of males (n=6, 9) and (**C’**) females (n=4, 11) after 12 weeks NC as assessed by EchoMRI. (**D**) Fat mass of males (n=6, 9) and (**D’**) females (n=4, 11) after 12 weeks NC as assessed by EchoMRI. (**E**) Fasting blood glucose in males (n=12) and (**E’**) females (n=12, 21) at 6 weeks NC.(**F**) Pancreas weight normalized to body weight (BW) post 12 weeks of HFD for males (n=15, 19) and (**F’**) females (n=12, 24). (**G**) Liver weight normalized to body weight (BW) post 12 weeks of HFD for males (n=15, 19) and (**G’**) females (n=11, 24). (**H**) Gonadal fat pad weight normalized to body weight (BW) post 12 weeks of HFD for males (n=13, 19) and (**H’**) females (n=12, 24). (**A** and **B**) were presented as mean ± SEM, two-way ANOVA. (**C**-**H**) were presented as mean ± SEM, unpaired t-test for males and one-way ANOVA for females. p values, *$p < 0.05$, ***$p<0.001$.

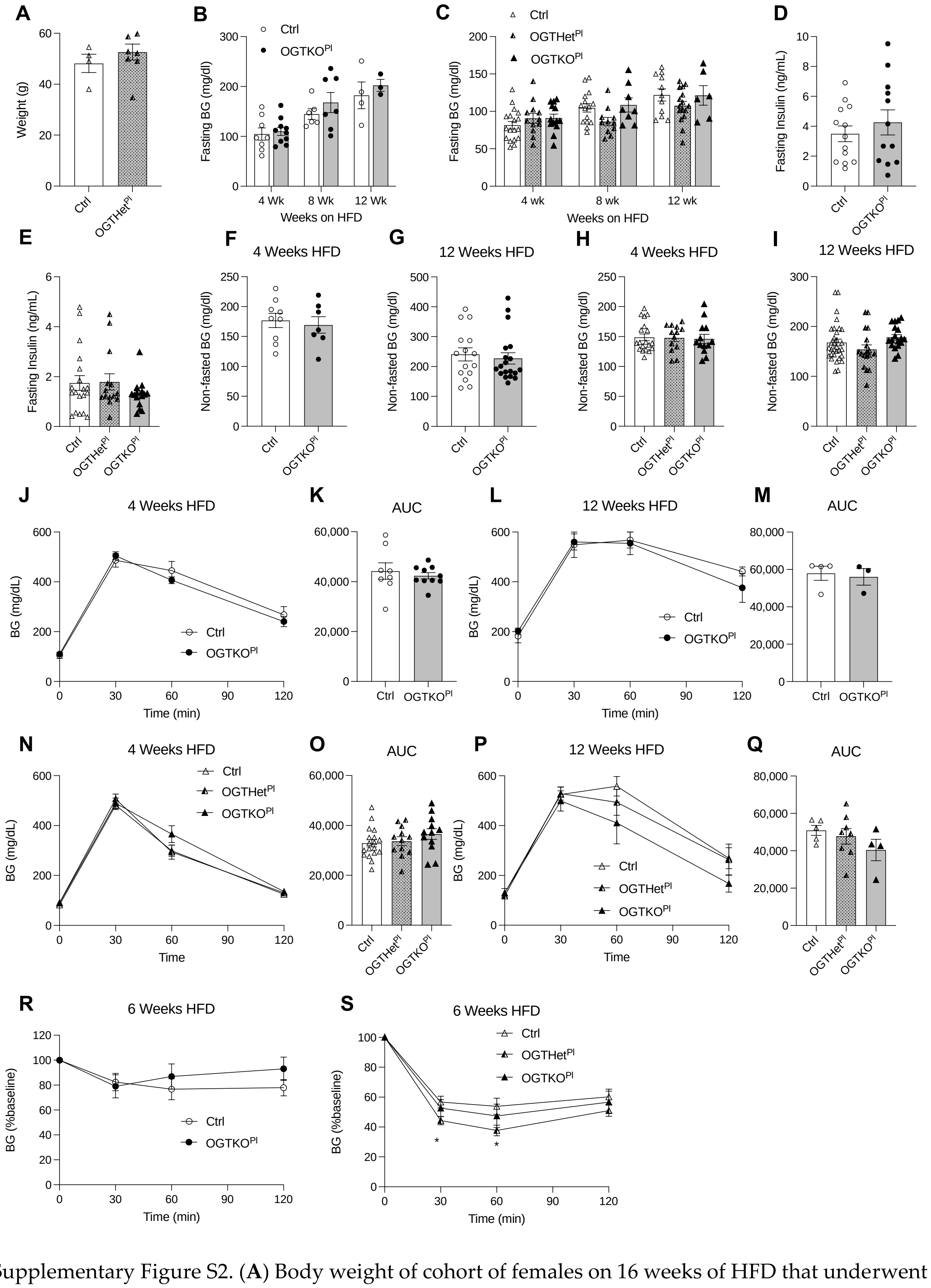

Supplementary Figure S2. (**A**) Body weight of cohort of females on 16 weeks of HFD that underwent indirect calorimetry testing (n=4, 8). (**B**) Fasting blood glucose was obtained at 4, 8 and12 weeks of HFD for males (n=3, 10) and (**C**) females (n=6, 18). (**D**) Fasting insulin levels at 12 weeks of HFD for males (n=12, 13) and (**E**) females (n=14, 19). (**F**) Non-fasted blood glucose for males was obtained at 4 (n=7, 9) and (**G**) 12 weeks (n=15, 19) of HFD. (**H**) Non-fasted blood glucose for females was obtained at 4 (n=13, 19) and (**I**) 12 weeks (n=18, 31) of HFD. (**J**) IPGTT and (**K**) calculated AUC at 4 weeks of

HFD for males (n=8, 10). (**L**) IPGTT and (**M**) calculated AUC at 12 weeks of HFD for males (n=3, 4). (**N**) IPGTT and (**O**) calculated AUC at 4 weeks of HFD for females (n=12, 18). (**P**) IPGTT and (**Q**) calculated AUC at 12 weeks of HFD for females (n=4, 8). (**R**) ITT at 6 weeks on HFD for male (n=7, 8) and (**S**) females (n=9, 19). Blood glucose values post insulin injection are expressed as a percent of baseline blood glucose. (**A**, **B**, **D**, **F**, **G**, **K** and **M**) were presented as mean ± SEM, unpaired t-test. (**C**, **E**, **H**, **I**, **O**, and **Q**) were presented as mean ± SEM, one-way ANOVA. (**J**, **L**, **N**, **P**, **R** and **S**) were presented as mean ± SEM, two-way ANOVA. p values, *$p < 0.05$.

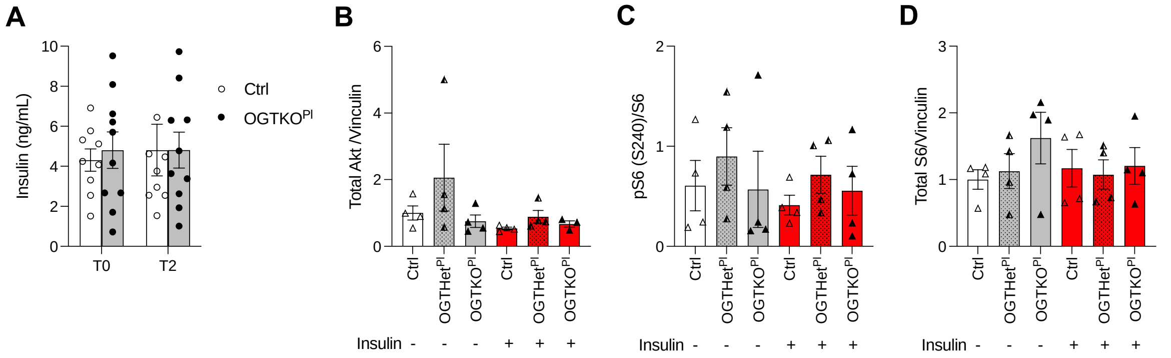

Supplementary Figure S3. (**A**) In vivo glucose stimulated insulin secretion (GSIS) for males (n=9, 10). T0 is a fasted state. T2 is two minutes post insulin injection. (**B**) Quantification of western blot for total Akt/Vinculin, (**C**) pS6(S240)/S6, and (**D**) total S6/Vinculin from liver samples visualized in (Figure. 5G). (**A**) was presented as mean ± SEM, two-way ANOVA. (**B-D**) were presented as mean ± SEM, one-way ANOVA.
